# Key Diagnostic Finding in a Condition with Variable Clinical Presentations

**DOI:** 10.1155/2013/415463

**Published:** 2013-08-05

**Authors:** Anju Sukumaran, John Buchlis

**Affiliations:** Department of Pediatric Endocrinology, Women and Children's Hospital of Buffalo, 219 Bryant Street, Buffalo, NY 14222, USA

## Abstract

This is an interesting case series on a very common genetic condition which are often diagnosed late as clinical signs are inconspicuous. We would like to highlight the principal clinical examination finding which led to diagnosis.

## 1. Cases

Certain common clinical entities have different clinical presentations. Though presenting features are varied, they can have key diagnostic findings in common. More often such patients are diagnosed late because these findings may be overlooked. We present 4 young men with disparate clinical presentations sharing a common final diagnosis. Their diagnostic findings are summarized in Table 1.

**Case 1**

A 16 year old male was referred to pediatric Endocrinology for short stature and low insulin like growth factor 1 (IGF-1) level. The subject was born full term and had age appropriate development. There was no family history of delayed puberty. Review of his growth chart showed that he tracked <3rd percentile for height and weight until the age of 15 years when there was height deceleration. Preliminary laboratory studies to evaluate short stature showed normal comprehensive metabolic panel and thyroid function. His IGF-1 level was 43 *μ*g/L (normal 187–599 *μ*g/L). Bone age was 13 years. At consultation, his height was at 4th and weight was <3rd percentile.

**Case 2**

A 16.5 year old male was referred for gynecomastia. The patient reported learning difficulties at school. Breast development was noted for the past 3 years, but there was no complaint of breast tenderness or nipple discharge. The patient was 187 cm tall (95th percentile) and was not obese. Laboratory testing revealed normal thyroid function, prolactin level, negative hCG, and elevated estradiol (201.8 pmol/L).

**Case 3**

A 19 year old male was referred for delayed puberty. Two years prior, the patient was evaluated by his primary physician for suspicion of a testicular mass. At that time, ultrasound examination measured right testes 2.3 × 1.1 × 2.1 cm and left testes 2.3 × 1.3 × 1.9 cm. At the referral, no family history of delayed puberty was elicited, and the patient stated that he was doing well in college.

**Case 4**

A previously healthy 17 year old male had a sudden onset of chest pain and dyspnea. He was diagnosed with mediastinal choriocarcinoma and received appropriate treatment. During the course of his evaluation, it was noted that he had small testes and was referred for further evaluation.

## 2. Final Diagnosis

In this case series, 4 young men presented to the endocrinologist with a variety of concerns—short stature, gynecomastia, delayed puberty, and mediastinal choriocarcinoma. Interestingly, they all had a common physical finding—small testicular size for their age with appropriate penile size. These patients all had elevated gonadotropins indicative of primary gonadal failure. The testosterone levels were in early to mid-pubertal range. Karyotype analysis was 47, XXY for all of the subjects, confirming a diagnosis of Klinefelter syndrome.

## 3. Clinical Course

Each subject was started on testosterone replacement therapy to treat the gonadal failure. Response (and adherence) to therapy was assessed by measuring serum testosterone levels, with the goal of achieving adult range testosterone (10−35 nmol/L) while monitoring for polycythemia. A DEXA scan was done at the beginning of treatment and was repeated every 1-2 years to monitor bone mineral accrual. The patient with gynecomastia underwent surgical resection of glandular breast tissue. The patient with mediastinal choriocarcinoma continued in remission.

## 4. Discussion

The first physical sign of gonadarche in boys is an increase in testicular size. Tanner staging is based on visual inspection and palpation. Measuring testicular length is an accurate and easier way of assessing the size of testis. A testis with a length of ≥ 2.5 cm indicates gonadotropin stimulation or central puberty [3]. At any stage of pubertal development, there may be a twofold range in testicular size. The range for penile size is 5-6 cm in prepubertal boys to 9–13 cm in adults. The average age of gonadarche is between 11 and 11.5 years. During gonadarche, there is a progressive rise in gonadotropins (LH and FSH) secretion, followed by increasing production of testosterone. Reference ranges for serum LH, FSH, and testosterone [1, 2] vary with genital staging and are dependent on the assay used.

Klinefelter syndrome (KS) was first described by Harry F. Klinefelter in 1942. He identified a clinical entity characterized by gynecomastia, small testes, normal to moderately reduced Leydig cell function, and increased secretion of FSH, indicative of absent spermatogenesis. 80% of patients with KS have the classic 47, XXY karyotype; the remaining 20% are mosaic (i.e., 46, XY/47, XXY) or have varying numbers of additional X and/or Y chromosomal material (i.e., 48, XXYY) [4]. KS represents the most common sex chromosome abnormality with an estimated prevalence of 1 in 600 live male births [5]. Other clinical features include reduced muscle strength, reduced facial and pubic hair, abdominal adiposity, metabolic syndrome, type 2 diabetes, and osteopenia [4, 6]. The clinical signs may be subtle and, hence, less than ten percent of children with KS are diagnosed before puberty [5, 7]. At birth, most 47, XXY infants appear normal. During childhood, they often present with speech delay, learning disabilities, or behavior problems [7, 8]. Clinically, an increase in height velocity may occur between ages 5 and 8 years due to greater leg growth; however, there is no difference in the timing and magnitude of pubertal growth spurt compared to unaffected males [9]. During puberty, testosterone production is adequate to support normal onset and development of secondary sexual characteristics [10]. However, from midpuberty onwards, KS subjects show gradual increase in FSH and LH levels to hypergonadotropic levels [10]. Testosterone replacement is necessary to treat androgen deficiency, support linear growth and development of secondary sexual characteristics, optimize bone mineral deposition, normalize fat distribution, and improve psychosocial well-being.

KS is one of the most common genetic causes of infertility in men. Testicular biopsies of prepubertal KS boys demonstrate preservation of seminiferous tubules with reduced numbers of germ cells; however, Sertoli and Leydig cells appear normal. Alternatively, the testes of the adult male with KS are characterized by extensive fibrosis and hyalinization of the seminiferous tubules as well as interstitial hyperplasia. Scattered throughout are some seminiferous tubules with residual foci of spermatogenesis. But introduction of testicular sperm extraction in combination with intracytoplasmic sperm injection techniques has allowed some KS males to father children.

Here we present 4 cases with unusual clinical presentations for KS.  Case 1 is unique because one of the classic characteristic features of KS is tall stature. Some uncommon variants of KS such as 49, XXXXY and isochromosome Xq are associated with short stature. Growth hormone deficiency has been reported in Klinefelter patients only in few cases. Our first patient had low IGF-1. His growth rate was calculated as 11.5 cm/year which was adequate for pubertal range. So growth hormone deficiency was unlikely. Normal growth spurt rate for boys is between 7 and 12 cm/year. He had normal adrenal hormones and negative hCG, ruling out other sources for androgen.

Case 2. Breast assessment should be a part of routine physical examination in boys. Pubertal gynecomastia is normal, occurring in at least two-thirds of boys in mid-puberty and usually regresses in one to two years. All cases of gynecomastia in prepubertal boys need to be further evaluated which was the basis of workup in our patient. KS patients are shown to have increased risk of developing breast cancer.

Case 3. The normal age range for first sign of puberty in males extends from 9 and 13.5 years. If a boy starts puberty before 9 years of age, it is considered precocious. Delayed puberty warrants investigation if there is no testicular change by 14 years of age or if it is taking more than 4 years for completion of puberty. The most common scenario for delayed puberty is constitutional growth delay, but 50–75% of patients will have a family history of delayed puberty. Our patient did not have any increase in testicular size even at the age of 17 years as shown by ultrasound report.

Case 4. Germ cell tumors are associated with KS. They manifest as precocious puberty in younger males and with mediastinal pressure symptoms in older children as we described here.

## 5. Conclusion

In summary, in all the cases described above, the main factor which led us to the diagnosis was examination of testes. If sexual maturity staging for penis and testes does not correlate, then puberty progression or alternate sources of androgen should be investigated. In Klinefelter syndrome, it is likely to have small firm testes during prepubertal years. There is an initial increase in volume at the onset of puberty. Size and consistency tend to become abnormal over time, resulting in decreased volume and firmer consistency. Accurate staging and followup of secondary sexual characteristics are important. Thus yearly testicular examination for age appropriate size and consistency is a very effective screening tool in diagnosing and managing gonadal failure.

## Figures and Tables

**Table 1:** Patient data.

Age of patient in years	Reason for referral	Pubertal staging			Laboratory evaluation		
		Testes Length in cm	Tanner Stage Pubic hair	Penis	FSH IU/L (normal range) [1]	LH IU/L (normal range) [1]	Testosterone nmol/L (normal range) [2]
Case 1: 16	Short stature	2	3	Adult	15.03 (0.16–3.7)	12.04 (0.25–2.7)	14.65 (<0.17)
Case 2: 16.5	Gynecomastia	3.2	4	Adult	37.5 (0.18–12)	28.2 (0.34–5.0)	11.24 (<5.79)
Case 3: 19	Delayed puberty	3	4	Adult	14.6 (0.18–12)	17 (0.34–5.0)	13.15 (<5.79)
Case 4: 17	Germ cell tumor and small testes	2.5	5	Adult	16.13 (0.18–12)	32 (0.34–5.0)	4.788 (<5.79)

Legend: testes <2.5 cm are prepubertal [3] and 2.5–3.2 cm represent first stage of puberty. Normal ranges of FSH, LH, and testosterone in parenthesis are 2.5th to 97.5th percentile values for corresponding testicular pubertal stage. Though all patients are of similar age range, their testicular pubertal stage is different and hence their normal testosterone levels are different.
